# 1215. A Mixed Methods Study on Severe Bacterial Infections in People Who Inject Drugs

**DOI:** 10.1093/ofid/ofab466.1407

**Published:** 2021-12-04

**Authors:** Alexander Hrycko, Benjamin Eckhardt, Pedro Mateu-Gelabert, Courtney Ciervo

**Affiliations:** 1 NYU School of Medicine, New York, New York; 2 NYU School of Medicine/Bellevue Hospital, New York, NY; 3 CUNY School of Public Health, New York, New York; 4 CUNY, New York, New York

## Abstract

**Background:**

Severe bacterial infections (SBI) associated with intravenous drug use have been increasing in frequency in the U.S. over the last decade. This mixed methods study aims to identify the risk factors associated with SBI in hospitalized individuals with recent injection drug use.

**Methods:**

We conducted 34 quantitative and 15 qualitative interviews between August 2020 and June 2021 at Bellevue Hospital in New York City. Eligible participants were (1) >/= 18 year of age, (2) admitted with a SBI, and (3) reported injection drug use within the 90 days prior to admission. Quantitative and qualitative data was obtained using a quantitative survey and in-depth, semi structured interviews of participants respectively. Analysis was performed to examine trends and explore common themes potentially contributing factors to SBI.

**Results:**

Of the 34 participants included, the median age was 37.5, 85% were male, 53% white, and 65% reported being homeless within the past 3 months. Endocarditis was the most common primary diagnosis (65%). Median length of hospital stay was 24 days and 35% required ICU level care during admission. A causative microorganism was identified in 85% of participants and 50% had *Staphylococcus aureus* as the sole organism. Discharges against medical advice occurred in 35%. Daily injection drug use in prior 30 days was 95% with a median of 10 injections per day. In the 30 days prior to admission, 50% reported an increase in injection frequency, 80% reported reusing needles and/or syringes, 75% reused cookers, 65% reused cottons. Analysis of qualitative interview data revealed high risk injection behaviors. Participants were not practicing and unaware of strategies to reduce their risk of drug injection-related SBI. Prior hospitalizations for SBI did not impact on this knowledge deficit on what constitutes bacterial infection risk and how to prevent it.

**Conclusion:**

Study findings highlight the complexity of the injection drug use process and the potential social and physiological pathways leading to SBI. Multiple domains at the structural, network, and individual level that impact drug injection practices and provide context by which these factors predispose and lead to physiological tissue damage and the development of SBI among PWID.

**Disclosures:**

**Benjamin Eckhardt, MD, MS**, **Gilead Sciences** (Grant/Research Support)

